# Aortic Valve Disease and Associated Complex CAD: The Interventional Approach

**DOI:** 10.3390/jcm10050946

**Published:** 2021-03-01

**Authors:** Federico Marin, Roberto Scarsini, Rafail A. Kotronias, Dimitrios Terentes Printzios, Matthew K. Burrage, Jonathan J. H. Bray, Jonathan L. Ciofani, Gabriele Venturi, Michele Pighi, Giovanni L. De Maria, Adrian P. Banning

**Affiliations:** 1 Oxford Heart Centre, Oxford University Hospitals, NHS Trust, Oxford OX3 9DU, UK; federicomarin@alice.it (F.M.); rafail.kotronias@cardiov.ox.ac.uk (R.A.K.); dimitrios.terentes-printzios@ouh.nhs.uk (D.T.P.); matthew.burrage@ouh.nhs.uk (M.K.B.); giovanniluigi.demaria@ouh.nhs.uk (G.L.D.M.); 2 Department of Cardiology, University of Verona, 37129 Verona, Italy; scarsini.roberto@gmail.com (R.S.); gabriele.venturi.vr@gmail.com (G.V.); michele.pighi@univr.it (M.P.); 3 Institute of Life Sciences 2, Swansea Bay University Health Board and Swansea University Medical School, SA2 8QA Swansea, UK; Jonathan.Bray@wales.nhs.uk; 4 Department of Cardiology, Royal North Shore Hospital, 2065 Sydney, Australia; ciofani.jc@gmail.com

**Keywords:** aortic stenosis, coronary artery disease, myocardial revascularization, percutaneous coronary intervention

## Abstract

Coronary artery disease (CAD) is highly prevalent in patients with severe aortic stenosis (AS). The management of CAD is a central aspect of the work-up of patients undergoing transcatheter aortic valve implantation (TAVI), but few data are available on this field and the best percutaneous coronary intervention (PCI) practice is yet to be determined. A major challenge is the ability to elucidate the severity of bystander coronary stenosis independently of the severity of aortic valve stenosis and subsequent impact on blood flow. The prognostic role of CAD in patients undergoing TAVI is being still debated and the benefits and the best timing of PCI in this context are currently under evaluation. Additionally, PCI in the setting of advanced AS poses some technical challenges, due to the complex anatomy, risk of hemodynamic instability, and the increased risk of bleeding complications. This review aims to provide a comprehensive synthesis of the available literature on myocardial revascularization in patients with severe AS undergoing TAVI. This work can assist the Heart Team in individualizing decisions about myocardial revascularization, taking into account available diagnostic tools as well as the risks and benefits.

## 1. Introduction

Aortic stenosis (AS) is the most common heart valve disease and is frequently associated with coronary artery disease (CAD). Recent multi-center trials report a high prevalence (~60%) of significant coronary stenosis among patients undergoing transcatheter aortic valve implantation (TAVI) [1,2]. The strong association between the two conditions is primarily due to an ensemble of clinical and genetic risk factors shared by both diseases including age, smoking, hypertension and hyperlipidemia [3]. The management of CAD is a central aspect of the work-up for TAVI, but the evidence available is still limited and the best percutaneous coronary intervention (PCI) practice in TAVI candidates is yet to be determined. A major challenge in patients with severe AS is the ability to elucidate the severity of bystander coronary stenosis independently of the severity of aortic valve disease. Furthermore, PCI in the setting of severe AS poses some technical challenges, including a high burden of complex and often heavily calcified coronary disease, the risk of hemodynamic instability and a potential series of challenges in re-engaging coronary ostia after TAVI requiring advanced and dedicated skills. An accurate selection of patients who need to undergo valve replacement plus myocardial revascularization is, therefore, paramount.

## 2. Anatomical CAD Assessment in AS

### 2.1. Coronary Angiogram

Coronary angiogram is the first line method for the assessment of coronary artery anatomy. According to the 2017 ESC Guidelines coronary angiography is recommended before valve surgery in patients with severe aortic stenosis and history of CAD, suspected myocardial ischemia, left ventricular dysfunction, one or more cardiovascular risk factor or in men older than 40-year old or postmenopausal women [4]. The coronary arteries of patients with severe AS are characterized by extensive calcification and tortuosity. Such characteristics reduce the reliability of angiography in the setting of AS, and in turn, hamper our ability to accurately estimate the degree of myocardial ischemia in TAVI patients [5]. In addition, there is only a modest correlation between angiography and intracoronary physiology in AS, especially in the territory of the left anterior descending artery (LAD), where even angiographically mild-moderate lesions may be functionally significant [6].

### 2.2. Coronary CT Angiography 

Contrast enhanced multi-detector computed tomography is pre-operatively performed to assess access route, and to assist with tissue heart valve sizing and other aspects of procedural planning. The role of computed tomography coronary angiography (CTCA) has been assessed and recently recommended for CAD evaluation in these patients, due to the practicality of acquiring ECG-gated coronary phases at the same setting without additional contrast [5].

When using invasive coronary angiography as a reference, the reported sensitivity and negative predictive value (NPV) of CTCA in identifying moderate obstructive CAD (>50% of diameter stenosis) in TAVI patients ranges between 90–100% and 90–96%, respectively (Table 1) [7,8,9,10,11]. Conversely, the specificity and positive predictive value (PPV) are often suboptimal and highly variable, ranging from 37–99%, and 37–95%, respectively (Table 1) [7,8,9,10,11]. A recent meta-analysis on 1275 patients undergoing CTCA and with a CAD prevalence similar to real world patients, showed 95% sensitivity, 65% specificity and 94% NPV; correctly identifying 442 (35%) patients as not having obstructive CAD [7]. The sub-optimal specificity can be largely explained by blooming artifacts and beam hardening due to the high calcium load that the coronary trees exhibit in TAVI candidates [7]. Rossi et al. have shown that increasing calcium score was associated with increased frequency of false positive and false negative results on CTCA and in the subgroup with <400 calcium score, CTCA had better diagnostic performance compared to ≥400 calcium score [12].

Nonetheless, a strategy using CTCA to rule out significant obstructive CAD may be still considered, with subsequent reductions in resource utilization and avoidance of exposing patients to the risks of invasive coronary angiography [18]. The outcomes of such a strategy were assessed in a cohort by Chieffo et al., showing that selection of patients following CTCA to undergo invasive coronary angiography either due to a suspicious coronary lesion (76%) or an uninterpretable CTCA (24%) was safe with no harm signal [17]. However, this study was retrospective and prone to selection bias and residual confounding despite attempts for adjustment [17].

## 3. Functional CAD Assessment in Aortic Stenosis

Symptom onset heralds a worse prognosis in AS and is a key indication for aortic valve replacement [4,19]. However, it is important to accurately determine whether symptoms are due to the valve itself, or co-existing CAD. The presence of chest pain is a poor discriminator, given the competing pathophysiology between hemodynamically significant CAD and severe AS. Angina is a typical prognostically significant symptom of severe AS and occurs frequently in the absence of obstructive CAD [20,21,22]. Angina in AS, in the absence of significant obstructive CAD is likely due to left ventricular hypertrophy, consequent increased oxygen demand and impaired myocardial perfusion. Microvascular dysfunction has been frequently described and may result from a combination of factors including increased resting flow and consequent reduced coronary flow reserve and extrinsic compression of the microvasculature [21,23,24]. Due to the propensity for ischemia, the specificity of non-invasive testing is lower in the presence of AS even without epicardial CAD [5].

### 3.1. Invasive Physiological Assessment 

The assessment of ischemic burden induced by a coronary plaque in presence of severe AS remains challenging since the most commonly used pressure-wire indices including fractional flow reserve (FFR) and instantaneous wave-free ratio (iFR) may be influenced by valve hemodynamics. The accuracy of FFR relies on the achievement of maximal hyperemia, which is normally obtained in the catheterization laboratory with the intravenous or intracoronary administration of vasodilatatory agents, such as adenosine. The complex interplay between the stenotic valve, elevated left ventricular end-diastolic pressure, left ventricular hypertrophy and the associated negative remodelling of the coronary microcirculation may blunt the response to adenosine and the achievement of maximal hyperaemia [25,26,27,28,29]. These factors may theoretically reduce the reliability of FFR in AS, causing a possible underestimation of the true ischemic significance of a given coronary obstruction.

Resting coronary flow is increased in patients with severe AS and this is the main pathophysiological element that affects coronary flow reserve (CFR) in this setting, reducing the delta between hyperemic and resting flow [30,31]. Stoller et al. investigated the changes in CFR and microvascular resistances before, and after, TAVI. Notably, resting flow estimated by transit time using thermodilution decreased significantly after TAVI. Conversely, hyperemic transit time remained unchanged after aortic valve replacement. Consistently, index of microcirculatory resistance (IMR) did not vary after TAVI (26 U vs. 30 U, *p* = ns) [31]. In a multicenter retrospective analysis, the delta between resisting Pd/Pa and FFR, a surrogate for hyperaemic microvascular vasodilation, was not significantly different between severe AS, moderate AS and controls, suggesting a clinically sufficient hyperaemic response to adenosine in patients with AS [32].

An opposite trend was observed by Ahmad et al. who recently performed doppler-derived coronary flow assessment after TAVI showing that resting flow do not change after TAVI whereas hyperemic flow increased significantly. Using wave intensity analysis, they were able to demonstrate that the coronary flow during the wave-free period, and consequently the iFR, were overall not influenced by TAVI. On the contrary, hyperemic flow increased significantly after TAVI with a consistent reduction in the mean FFR value [33]. Given the discordant results of the available data, the dilemma of coronary physiology in AS remains difficult to solve. However, most of the studies are concordant in identifying four main factors that should be taken into account when interpreting the results of a physiological test in AS: (1) Resting coronary flow can be increased; (2) peak hyperaemic coronary flow can be reduced; (3) the delta between baseline flow at maximal achievable coronary flow at rest can be reduced; (4) microvascular vasodilatation can be impaired by extravascular compression forces related to the increased LV end-diastolic pressure and remodeling associated with hypertrophy and fibrosis. 

The physiological alterations caused by aortic valve obstruction have recently been investigated by Yamanaka et al. comparing nuclear myocardial perfusion imaging and coronary physiology in severe AS patients. Notably, they observed a different behavior of both FFR and iFR in AS compared with standard stable CAD. In particular, the best cut-off for FFR was slightly increased compared with the standard clinical threshold (0.82 vs. 0.80) [34]. On the contrary the iFR best cut-off in detecting ischemia was significantly lower in AS (0.82 vs. 0.89). Lowering the iFR cut-off for detecting ischemia was also reported by other groups of investigators and it could be used to reduce unnecessary CAD treatment in TAVI candidates [35,36,37,38] (Table 2).

Other novel non-hyperemic pressure-wire indices may have the potential to be used to functionally assess coronary lesions in patients undergoing TAVI. However, no data are available so far and this represents an interesting field for future research [39].

### 3.2. Does Coronary Physiology Assessment Vary after TAVI?

To date, few studies have investigated the variations of physiological indices before and after aortic valve replacement. Pesarini et al. observed no overall significant variations in FFR measurement immediately before and after TAVI. Notably, a different trend was observed for coronary lesions with abnormal FFR at baseline compared to those lesions with FFR >0.80. In borderline or FFR-positive lesions, FFR tended to worsen after TAVI (0.71 ± 0.11 vs. 0.66 ± 0.14). Conversely, in FFR-negative lesions, FFR improved significantly (0.92 ± 0.06 vs. 0.93 ± 0.07). Overall, only 8 out of 133 lesions crossed the clinical 0.80 cut-off after TAVI [40]. Stoller et al. observed an improvement of FFR values after TAVI (0.90 ± 0.08 vs. 0.93 ± 0.08, *p* = 0.002), and this was related primarily to a significant decrease in hyperaemic mean aortic pressure [31]. A contemporary quantitative meta-analysis of 5 studies evaluating 250 coronary vessels in 169 patients with severe AS suggested that FFR in diseased vessels is not affected by TAVI, with a mean difference −0.01, 95% CI −0.03–0.01, *p* = 0.49 [41].

The variations of iFR before and after aortic valve replacement have been investigated by Scarsini et al. in a cohort of patients undergoing TAVI [42]. Overall iFR did not vary significantly before or after TAVI. However, threshold crossover was observed in up to 15% of the lesions after TAVI, shifting the indication for treatment more often compared to what observed for FFR-guided revascularization strategy [42]

Scarce data is available on the long-term variations of coronary physiology in patients undergoing aortic valve replacement. An exploratory study by Scarsini et al. in 23 coronary lesions showed that FFR decreased in 3(13%) lesions with abnormal baseline value, whereas it remained stable in lesions with FFR > 0.80; conversely, iFR did not show a systematic trend at long-term after TAVI and iFR demonstrated a higher reclassification rate at follow-up compared with FFR (*p* = 0.02) [43]. 

Conversely, Vendrik et al. reported a significant reduction of FFR values over time up to 6 months after TAVI, whereas iFR did not show significant variations at 6 months of follow up [44]. The authors concluded that using resting indices may be more appropriate to select TAVI candidates who require CAD treatment. Camuglia et al. described the variations of CFR in a small cohort of patients undergoing TAVI. CFR was measured in 8 severe AS patients at baseline, immediately after TAVI and 12 months later. Notably CFR was severely impaired at baseline in all the cases (0.65, 95% confidence interval (CI) 0.36–0.93) [45]. No significant variations were observed immediately after TAVI, a trend confirmed by Stoller et al. in a larger TAVI cohort [31]. Nonetheless, at 12-month follow up, CFR was significantly increased (2.18, 95% CI 1.88–2.47, *p* < 0.01) [45]. 

In summary, despite physiological assessment is safe and feasible in patients with untreated AS, the physiological alterations caused by the valvular obstruction can significantly impair the results. In particular, caution should be taken in the interpretation of borderline values, which should be reassessed after valve implantation.

### 3.3. Quantitative Flow Ratio in Presence of Aortic Stenosis

Quantitative flow ratio (QFR) (QAngioXA-3D prototype, Medis, Leiden, the Netherlands) is an angiography-based physiology software that uses thrombolysis in myocardial infarction (TIMI) frame count of a single-vessel in two orthogonal views as the surrogate marker of blood flow to calculate the translesional gradient ratio [46,47,48,49]. The diagnostic performance of QFR has been recently evaluated in patients with AS undergoing TAVI. A study performed by Hernán Mejía-Rentería et al. has enrolled 115 patients with severe AS and concomitant CAD (138 coronary arteries) who underwent FFR assessment before TAVI. The authors compared the diagnostic yield of post-hoc QFR using FFR as a reference and found that using ≤0.80 as cut-off for both techniques, QFR correctly classified the functional significance of coronary stenosis in 112 vessels (81%) [50]. Moreover, the authors showed that in patients with an aortic valvular area (AVA) ≥0.80 cm^2^, the classification agreement between both methods was as high as 91%, and it decreased to 79% when AVA was 0.60–0.80 cm^2^, and to 66% when AVA was <0.60 cm^2^ (*p* = 0.022 for comparison between AVA ranges) [50]. Notably the diagnostic yield of QFR in this setting is lower than reported in other settings and could be related to the presence of microvascular impairment in accordance with previous studies [50], but further studies are needed.

### 3.4. FFR_CT_ in Presence of Aortic Stenosis

Fractional flow reserve-computed tomography (FFR-CT) uses computational flow dynamics to simulate invasive FFR from a standard CTCA acquisition, providing both anatomical and functional information. Michail et al. has recently assessed the feasibility, and validity of FFR-CT in 42 patients with AS, who underwent both CTCA and conventional FFR measurement with a pressure wire (CAST-FFR study) [51]. The authors reported a strong correlation between conventional pressure wire-derived FFR and FFR-CT (AUC 0.83; 0.72–0.93, *p* < 0.0001) and the sensitivity, specificity, PPV and NPV were 73.9%, 78.4%, 68.0%, and 82.9%, respectively, with 76.7% diagnostic accuracy [51]. To assess the validity of FFR-CT in patients with AS will be the aim of the FORTUNA (Evaluation of Fractional Flow Reserve Calculated by Computed Tomography Coronary Angiography) trial (Clinicaltrial.gov: NCT03665389) which is a single-center, open-label, exploratory, prospective study, that will compare iFR pre-TAVI, FFR- and iFR-post TAVI with the corresponding FFRCT measurements pre and post TAVI.

## 4. Impact of Myocardial Revascularization in Patients Undergoing TAVI 

**Figure 1:**
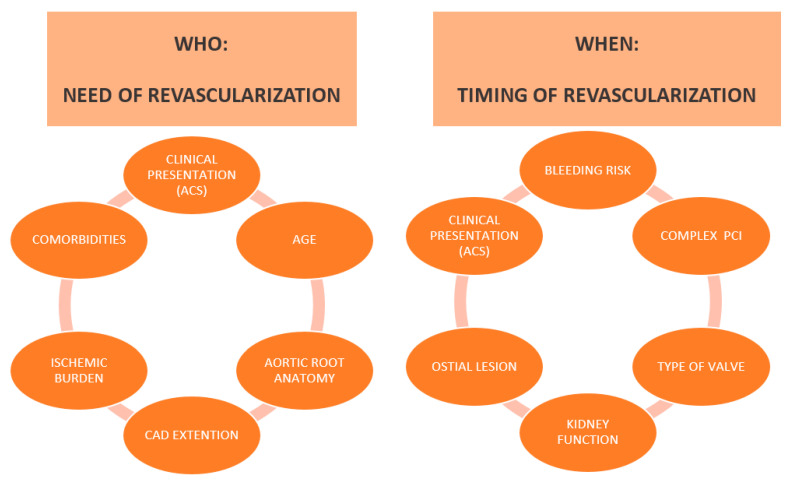
The management of CAD in TAVI patients: who need myocardial revascularization and when? Abbreviations: ACS, Acute coronary syndrome; PCI, Percutaneous coronary intervention; CAD, Coronary artery disease.

In surgical candidates, the current guidelines suggest that myocardial revascularization with coronary artery bypass graft (CABG) at the time of surgical aortic valve replacement (SAVR) is a class I recommendation in the presence of stenoses of ≥70%, and a class IIa recommendation if the stenoses are 50–70% on angiography. This recommendation is based on small, non-randomized, retrospectives studies, which showed that non-revascularised CAD patients have poorer 10-year survival rates than those undergoing revascularization or not requiring CABG [52,53]. Moreover, it is reasonable to combine the two procedures (coronary revascularization and valve replacement) whenever a major cardiac operation is planned in light of the risks related with a future redo operation. A recent meta-analysis by Kotronias et al. showed that a percutaneous transcatheter approach combing TAVI and PCI conferred similar outcomes to a surgical approach combining SAVR and CABG [54]. Therefore, patients with severe AS and CAD can have either approach determined predominantly by the surgical risk. Indeed, the 2017 ESC/EACTS Guidelines for the management of valvular heart disease suggest that PCI should be considered in the presence of coronary artery diameter stenosis >70% in proximal segments (Class IIa) with a level of evidence C [4]. As acknowledged in the guidelines, limited evidence is available and the prognostic value of bystander CAD in patients with AS remains to be established, while the potential benefits of PCI in this setting remains unclear (Figure 1).

### 4.1. Prognostic Impact of CAD in Patients Undergoing TAVI 

The prognostic impact of CAD in patients undergoing TAVI is predominantly based on retrospective, single center studies, with small sample size and without a standardized definition of CAD and reported discordant results (Table 3). Some studies have demonstrated that CAD is associated with impaired clinical outcome. Dewey et al. have shown that patients with CAD had a 10.1-fold greater risk of mortality (95% CI: 2.1 to 174.8) within 30 days after TAVI procedure than those who did not. [55]. These results were further corroborated by data from the Bern TAVI and PCI registries which document an increased risk of ischemic events and cardiovascular mortality at 1-year follow-up (hazard ratio (HR)1.86, 95% CI 1.03–3.36, *p* = 0.040) [56]. Moreover, Stefanini et al. demonstrated that patients with a high preoperative SYNTAX score (Synergy Between PCI With Taxus and Cardiac Surgery, (SS)), defined as SS >22, had worse outcomes compared to the group with low SS [57]. This finding is in agreement with results from a large multi-centre study of 1270 TAVI patients that identified the same threshold of SS >22 as an independent predictor of all-cause mortality (HR 2.09; *p* = 0.017) [58]. In contrast, other studies have shown neutral impact of CAD when adjusting for mortality-modifying comorbidities. The German TAVI registry showed a higher crude in-hospital mortality in patients with CAD (10.0 vs. 5.5 %, Odds ratio (OR) 1.90, 95 % CI 1.23–2.93), which was no longer significant after adjustment for confounders (adjusted OR 1.41, 95 % CI 0.85–2.33) [59]. Similar findings have been obtained by the analysis of 2588 consecutive patients from the U.K. TAVI Registry which showed that after adjusting for confounders, the presence and extent of CAD was not associated with early (30-days, *p* = 0.36) or late (4 years, *p* = 0.10) survival [60]. Also, in a large meta-analysis including 2472 patients, it has been observed that CAD was not a risk factor for higher mortality (OR 1.0, 95% CI 0.67–1.50) [61]. 

### 4.2. Benefits of PCI in Patients Undergoing TAVI 

Despite the feasibility of PCI in severe AS patients scheduled for TAVI, the benefits of revascularization compared to optimal medical therapy remain uncertain. Previous studies comparing TAVI plus PCI versus TAVI alone have showed discordant results even when the degree of revascularization, assessed by residual SYNTAX score, was taken into account, as summarized in Table 3.

A meta-analysis conducted by Kotronias et al., including nine observational studies and 3858 patients, showed that severe AS patients that underwent revascularization with PCI had a higher rate of major vascular complications (OR 1.86; 95% CI 1.33–2.60; *p* ≤ 0.001) and higher 30-day mortality (OR 1.42; 95% CI 1.08–1.87; *p* = 0.01) [62]. Moreover, there were no differences in effect estimates for 30-day cardiovascular mortality (OR: 1.03; 95% CI, 0.35–2.99), myocardial infarction (OR: 0.86; 95% CI, 0.14–5.28), acute kidney injury (OR: 0.89; 95% CI, 0.42–1.88), stroke (OR: 1.07; 95% CI, 0.38–2.97), or 1-year mortality (OR: 1.05; 95% CI, 0.71–1.56) [62]. This finding was further corroborated by the presentation of the preliminary results of the (The percutAneous Coronary inTervention prIor to transcatheter aortic VAlve implantation) ACTIVATION trial [63]. The ACTIVATION trial is [64] a prospective, multicenter study which randomized a total of 235 patients with at least 1 lesion of ≥70% severity in a major epicardial vessel to PCI versus conservative management prior to TAVI. The authors reported no difference in the primary endpoint of death and rehospitalization at one year (41.5% in the PCI group vs. 44% in the no-PCI group, *p* = 0.067) [63]. Additionally, the study reported a higher rate of bleeding in the PCI group (44.5% vs. 28.4%, *p* = 0.02) [64]. Notably, patients with history of active bleedings, recent acute coronary syndrome, left main disease, or class III-IV angina were excluded from the study. 

The absence of prognostic benefit of PCI in severe AS patients undergoing TAVI is consistent with the findings of the literature on myocardial revascularization in stable CAD [65,66]. 

It is the opinion of the authors that a stratified approach is required to identify severe AS patients who will symptomatically and/or prognostically benefit from PCI. Further research into biomarkers and clinical pathways is required to identify patients who may benefit the most from CAD revascularization. This is particularly important as TAVI is expanding to younger and lower risk severe AS patients with a longer life-expectancy than the high-risk severe AS patients the current body of research has so far focused on [67,68]. 

### 4.3. FFR Guided Revascularization

Current ESC guidelines on myocardial revascularization strongly recommend physiological assessment of borderline coronary lesions in patients with stable CAD [69]. On the other hand, recent ESC guidelines on management of valvular heart disease, recommend to assess CAD in patients with AS undergoing aortic valve replacement by means of angiography alone [4]. The reason for this disagreement lies in the complex pathophysiology of coronary flow in AS and the consequent exclusion of these patients from any validation or outcome trial on physiological indices [70,71,72,73,74]. Up to date, few data are available on the clinical outcome of FFR-guided revascularization in patients undergoing TAVI. When coronary physiology is used to guide the myocardial revascularization of patients with severe AS, it leads to an important simplification of the clinical management. Di Gioia and colleagues demonstrated that FFR allows to downgrade the number of diseased vessel compared to coronary angiography alone without increasing the adverse event rates up to 5 years (38% vs. 39%; *p* = 0.98) [75]. In a retrospective observational study including 216 patients undergoing TAVI, Lunardi et al. demonstrated that FFR-guided revascularization presented a better major adverse cardiac and cerebrovascular events (MACCE)-free survival at 24 months compared with those who underwent angiography-guided revascularization (92.6% versus 82.0%; *p* = 0.035). Bystander intermediate coronary lesions were FFR negative in 78.2% of cases and were safely deferred without ischemic complications during the TAVI procedure and long term [76]. To confirm these observations, a nationwide, randomized clinical trial (FAITAVI (Functional Assessment in TAVI), Clinicaltrial.gov: NCT03360591) is currently ongoing to compare FFR-guided versus angiography-guided revascularization in patients undergoing TAVI. Similarly awaited are the results of the ongoing Revascularization in Patients Undergoing Transcatheter Aortic Valve Implantation (NOTION-3) trial, comparing an FFR-guided complete revascularization with PCI versus conservative management in patients undergoing TAVI (Clinicaltrial.gov: NCT03058627). 

In summary, the prognostic impact of CAD in patients with severe AS is still debated and the benefits of myocardial revascularization in patients with severe AS undergoing TAVI should be carefully evaluated by the Heart Team. The available literature recommends against routine revascularization in patient undergoing TAVI, especially in the presence of stable CAD. A multidisciplinary individualized decision should take into account patient’s age, comorbidities, the amount of myocardium at risk, and the clinical presentation. Myocardial revascularization should be considered in presence of disease involving the proximal segment of large epicardial vessel, especially in patients presenting with high-risk acute coronary syndrome where ischemia is mainly driven by plaque instability and in patients with refractory angina, even after relief of the AS. 

## 5. Timing of Percutaneous Coronary Intervention 

When coronary revascularization is indicated the optimal timing in relation to the valve intervention remains to be established and no randomized data are available (Table 4). The most widely adopted approach is to perform PCI before the implantation of the valve. Nevertheless, several studies have shown the safety and feasibility of performing both interventions during the same procedure or deferring PCI after the valve replacement.

### 5.1. PCI Upstream to TAVI

TAVI at its nascency was a complex and high-risk procedure that required prolonged cardiac pacing that could induce severe hypotension and possibly worsening of myocardial ischemia in patients with CAD. Moreover, there was very limited operator experience and there were very few studies examining the feasibility of coronary cannulation following a TAVI device implantation. Due to these concerns, the most common approach has been to routinely treat significant epicardial coronary disease before valve implantation. Despite the wide adoption of this approach, it should be acknowledged that it is not supported by robust clinical evidence. Nonetheless, two important considerations should be taken into account: the risk of performing complex PCI to achieve revascularization in patients with severe AS and the theoretical increase in bleeding risk during TAVI due to the requirement for dual anti-platelet therapy following coronary stent implantation. 

Limited data is available on the safety of PCI in patients with severe AS. Goel et al. showed that PCI can be performed in patients with severe symptomatic AS and CAD without an increased risk of short-term mortality compared with propensity-matched patients without AS. Nevertheless, the subgroup of patients with ejection fraction ≤30% or Society of Thoracic Surgeons score ≥10% have a significant higher 30-day mortality (respectively 5.4% versus 1.2%; *p* < 0.001 and 10.4% versus 0%; *p* < 0.001) [97]. In this subgroup of patients, who frequently undergo complex PCI requiring rotational atherectomy, adjunctive balloon aortic valvuloplasty and/or mechanical circulatory support could improve the safety profile of PCI in case of a procedural complication [98]. Kotronias et al. demonstrated that rotational atherectomy can be performed safely in patients with severe AS to modify highly calcified coronary vessels in preparation for stent implantation. Notably, the rate of complications was low and not significantly different when compared to the complications rate of a matched cohort of patients without AS [99]. Nevertheless, intraprocedural coronary complications may have a profound hemodynamic impact in the presence of untreated severe AS and require emergent rescue valve intervention (most case rescue aortic-balloon valvuloplasty) [100].

Another potential disadvantage of performing PCI before TAVI is the requirement of dual antiplatelet therapy after stent insertion that could carry an additional periprocedural hemorrhagic risk. Van Rosendael et al. investigated the clinical outcome of patients undergoing PCI within 30 days or >30 days before TAVI, showing a significant increase in minor vascular injury and bleeding complications after TAVI in the group who had PCI within 30 days before TAVI [101]. 

In summary, staged PCI before TAVI should be considered when the risks of untreated myocardial ischaemia outweigh the risks of valve replacement in itself. It is reasonable to consider PCI before TAVI in patients with acute coronary syndrome and a large area of myocardium at risk due to plaque instability, patient with critical ostial lesions which may increase the risk of coronary occlusions during TAVI or in patients with concerns regarding future access to the coronary ostia due to unfavourable aortic anulus anatomy or in case of valve-in-valve procedure. In patients with indication to revascularization but at a very high risk of intra-procedural complications, adjunctive balloon aortic valvuloplasty plus/minus mechanical circulatory support could be considered to improve the safety of the PCI [98]. Notably, a novel non-occlusive balloon for balloon aortic valvuloplasty (TrueFlow™, BARD, Peripheral Vascular, Tempe, AZ, USA) has been recently approved which enable anterograde perfusion through the inner lumen during inflation and does not require rapid ventricular pacing [102].

### 5.2. TAVI Upstream to PCI

The strategy of performing TAVI upstream to PCI has not been largely investigated in the past due to concerns related to the safety of performing the valve implantation in patient with untreated coronary lesions. However, the improvement in technological equipment together with accurate pre-procedural planning has decreased the demand of pacing and also has reduced the overall risk of the procedure itself, allowing for the possibility for the interventionalist to safely defer coronary revascularization until after valve implantation.

**Figure 2:**
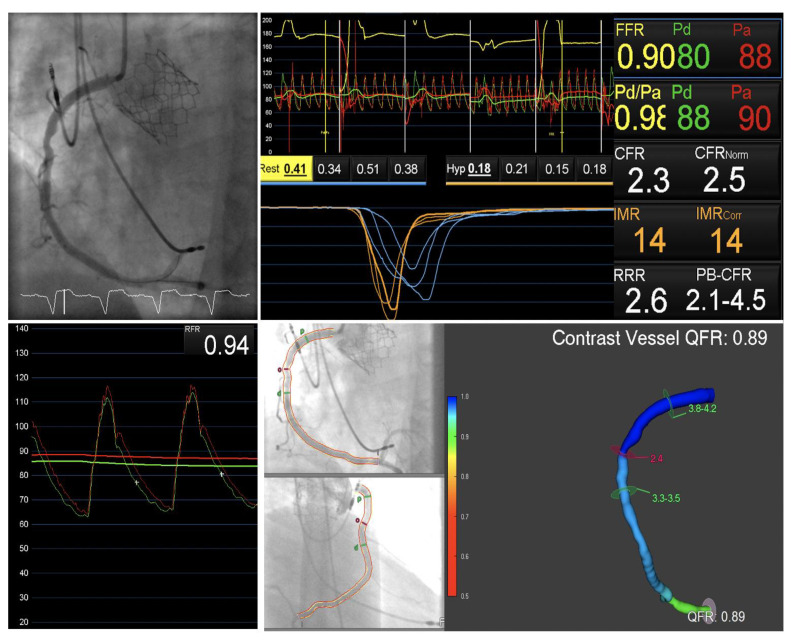
Functional assessment after TAVI implantation. Explanatory case showing the feasibility of performing functional assessment after valve implantation (Sapien 3, 26 mm, Edwards Lifesciences, Irvine, CA, USA) in a patient with moderate atheroma on the right coronary artery. This approach has the advantage of avoiding misleading interpretation of physiological indices in presence of Scheme 0. IMR was 14 U suggesting non inducible ischemia and preserved microvascular resistance. Below: RFR, a non-hyperemic full cycle pressure-wire based index, was 0.94 and three-dimensional (3D) quantitative coronary analysis and subsequent QFR computation was 0.89. Abbreviations: FFR, fractional flow reserve; QFR, quantitative flow ratio; IMR, index of micro-vascular resistance; RFR, resting full cycle ratio.

Deferring myocardial revascularization until after valve implantation is advantageous as it can allow symptom evaluation and residual ischemic burden assessment free from the confounding effect of severe AS (Figure 2). Since TAVI is moving toward younger patients at intermediate-to-low surgical risk, the appropriate identification of patients who could yield maximal symptomatic and prognostic benefit is of paramount importance.

**Figure 3:**
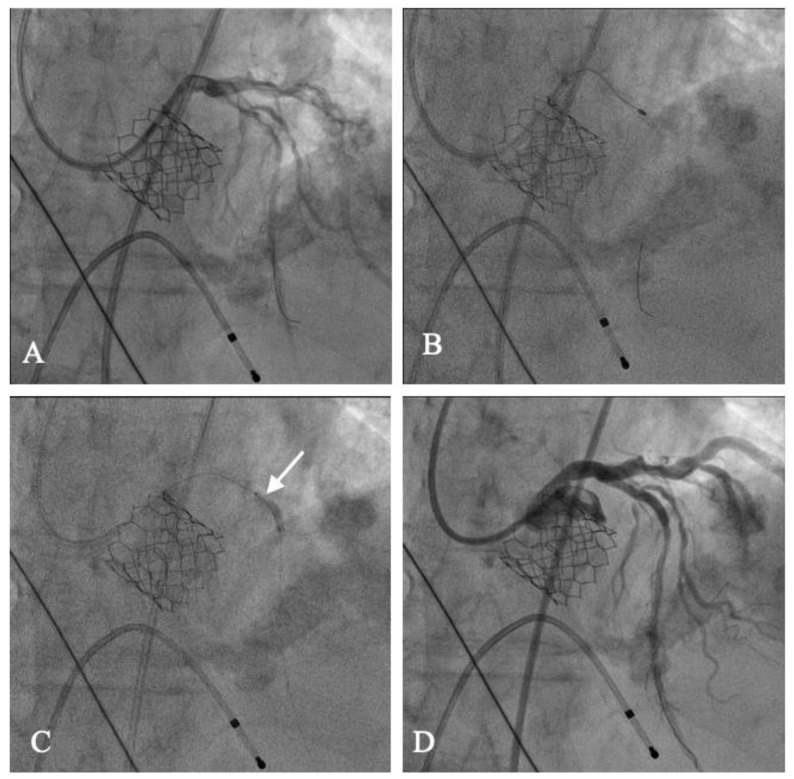
PCI with rotational atherectomy and stenting after TAVI (Edwards Sapien 3). (**A**) Coronary angiography after selective cannulation of left coronary artery showed severe calcific lesion of the mid left anterior descending. (**B**) Rotational atherectomy. (**C**) Predilation of the lesion with non-compliant balloon. (**D**) Final result after stent implantation.

Another potential advantage of an approach based on performing TAVI upstream is to improve the hemodynamic performance of the left ventricle prior to PCI. Following relief of AS, myocardial afterload decreases, as well as myocardial oxygen consumption and cardiac output significantly increases with subsequent improvement of systemic perfusion [103]. Several studies have shown an improvement both of systolic and diastolic function of the left ventricle after TAVI [104]. The haemodynamic improvement may increase the safety of performing complex PCI, which due to high calcium burden, may require extensive lesion preparation with rotational atherectomy and prolonged balloon inflation in proximal segments of the coronary tree (Figure 3) [105,106]. Moreover, the impact of contrast administration on kidney function in patients who had undergone TAVI may be better tolerated because of the hemodynamic changes following aortic valve replacement [107]. Recently, Venturi et al. reported that contrast-induced acute kidney injury occurred less frequently in patients undergoing TAVI than in patients without AS undergoing PCI, despite a worse-risk profile (OR 0.33, 95%CI 0.19–0.58, *p* = 0.002). It remains to be elucidated if the timing of PCI (before TAVI versus after TAVI) may have an impact on residual renal function in patients undergoing TAVI.

**Figure 4:**
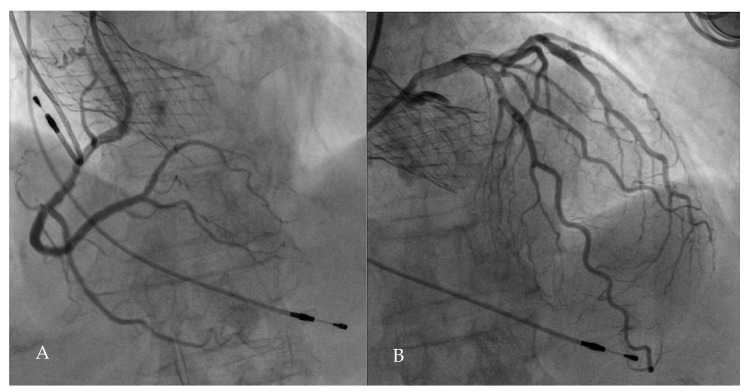
Feasibility of coronary access post TAVI. Coronary angiography performed after TAVI thorough the CoreValve Evolut R struts. (**A**) Right coronary artery selective cannulation using a Judkins right catheter (JR 4, Cordis Corporation, Bridgewater, NJ, USA); (**B**) Left coronary artery selective cannulation with an extra backup support catheter (EBU 3.5, Medtronic Inc., Minneapolis, MN, USA).

A concern about performing PCI after TAVI is the technical challenge of coronary cannulation and catheter manipulation in the presence of a TAVI prosthesis (Figure 4). The ability to access coronary ostia depends on anatomical factors such as sino-tubular junction height and width and coronary height but also on the type of prosthesis and height of implantation. Self-expanding valves have a supra-annular position above the coronary ostia that could impede coronary access especially when a neocommissure lies in front of the coronary ostium. Balloon-expandable valves have a shorter height compared to the self-expandable valves. Moreover, the stent-cells of the upper row are larger, allowing easier access to the coronary ostia. The feasibility of coronary ostia cannulation after TAVI has been recently investigated by the RE-ACCESS (Reobtain Coronary Ostia Cannulation Beyond Transcatheter Aortic Valve Stent) study [108]. This single-center, prospective, registry-based study enrolled 300 consecutive patients undergoing TAVI using all commercially available devices and undergoing coronary angiography before, and after, TAVI. The authors found a total of 23 (7.7%) cases of unsuccessful coronary cannulation after TAVI, and this issue occurred in 22 of 23 cases with the use of Evolut R/PRO (Medtronic, Minneapolis, MN, USA) transcatheter aortic valves (17.9% versus 0.4%, *p* < 0.01) [108]. At multivariate analysis the combination of use of the Evolut valve, sinus of Valsalva oversizing, and depth of implantation had an excellent discrimination capability to predict unsuccessful coronary cannulation after TAVI. To account for this possible issue, technical refinements of TAVI implantation aimed at commissural alignment have been proposed. Tang et al. evaluated the impact of initial deployment orientation of the SAPIEN 3 (Edwards Lifesciences, Irvine, CA, USA), Evolut, and ACURATE-neo (Boston Scientific, Marlborough, MA, USA) TAVI valves on their final orientation and neocommissural overlap with coronary arteries [109]. The authors found that the initial SAPIEN 3 orientation had no impact on alignment, whereas specific adjustment in orientations of the Evolut and ACURATE-neo improved alignment [109]. Optimizing valve alignment to avoid overlap between neo-commissures and coronary ostia is essential to grant coronary artery access in case of future redo TAVI or need for PCI.

### 5.3. PCI and Concomitant TAVI

Whatever the chosen approach (TAVI or PCI first) several studies have shown the feasibility and safety of performing the two procedures within the same session. The potential advantages of this approach are: (i)No requirement for additional vascular access, (ii) lower theoretical risk of vascular complications, (iii) lower patient’s inconvenience and discomfort and iv) reduction in healthcare resources utilization. Ochiai et al. found that the timing of PCI either before TAVI (*n* = 143), concomitantly with TAVI (*n* = 77), or until after TAVI (*n* = 38) was not associated with 2-year major adverse cardiac and cerebrovascular events rate (concomitant vs. pre-TAVI, HR: 0.92; 95% CI: 0.52 to 1.66; *p* = 0.79; post- vs. pre-TAVI, HR: 0.45; 95% CI: 0.18 to 1.16; *p* = 0.10) [110]. Wenaweser et al. showed that the clinical outcome at 30 days was similar for patients undergoing isolated TAVI as compared with TAVI combined with PCI in terms of death (5.6% vs. 10.2%, *p* = 0.24), major stroke (4.1% vs. 3.4%, *p* = 1.00), and the VARC combined safety endpoint (31.0% vs. 23.7%, *p* = 0.33) [111]. More recently, Barbanti et al. confirmed that patients undergoing TAVI and PCI in the same setting had similar rate of the composite of death, disabling stroke, and myocardial infarction when compared with patients without CAD, and patients with severe CAD left untreated (TAVI + PCI: 10.4%; severe CAD left untreated: 15.4%; no-CAD: 14.8%; *p* = 0.765) [112].

A possible disadvantage of concomitant TAVI and PCI strategy is the amount of contrast media administered at the time of the procedure. Penkalla et al. noticed that radiation time and the amount of contrast agent were higher during combined treatment compared to a staged PCI strategy. This could translate into a higher risk of acute kidney injury, especially in patient with chronic renal disease, undergoing complex PCI. A recent meta-analysis, based on observational data comparing the short-term safety outcomes of concomitant versus staged PCI with TAVI in severe AS patients, demonstrated that the 30-day all-cause mortality and other major safety endpoints did not significantly differ between the two approaches [113]. Notably, the pooled rate of renal failure was not statistically different between the two groups, although the incidence was relatively higher in the concomitant PCI and TAVI group (5% versus 2.2%) [113]. Another disadvantage of combining two procedure in the same session is the increased of the procedural complexity as well as the operator fatigue.

## 6. Conclusions

Coronary artery disease is highly prevalent in patients with severe AS and frequently poses a clinical management challenge. The complex pathophysiology of coronary flow in AS makes the interpretation of symptoms, as well as the assessment of myocardial ischemia challenging. Although the prognostic impact of CAD in patients undergoing TAVI is still debated, the treatment of angiographic significant stable coronary stenosis with PCI seems to have no prognostic benefits. Conversely treatment of unstable coronary disease is still likely to retain a clinical benefit in candidates to TAVI. The benefits and timing of myocardial revascularization in patients with severe AS undergoing TAVI should be carefully evaluated by the Heart Team and decisions individualised. Going forward, a stratified medicine approach will likely be required to identify severe AS patients who will symptomatically and/or prognostically benefit from PCI. Since TAVI is moving towards younger and lower risk patients, further research into biomarkers and clinical pathways is warranted to identify patients with severe AS who may benefit the most from myocardial revascularization.

## Figures and Tables

**Table 1:** The role of CTCA in TAVI candidates.

Study	Study Design	Patients (*n*)	Definition Significant Stenosis	Vessels Evaluated	Sn (%)	Sp (%)	PPV (%)	NPV (%)
Strong et al. [13]	Retrospective cohort	200	>50% stenosis	Native	100	42	48	100
Chaikriangkrai et al. [14]	Systematic review and meta-analysis	1498	>50% stenosis	All	96	74	–	–
Andreini et al. [8]	Prospective cohort	325	>50% stenosis	Native (no stents)	91	99	8	100
				Native (stented)	94	87	67	98
				CABG	90	91	81	95
Annoni et al. [15]	Prospective cohort	115	–	All	97	85	62	99
Rossi et al. [12]	Prospective Cohort	140	>50% stenosis	All	91	55	59	90
			>70% stenosis	All	78	74	37	95
Matsumoto et al. [11]	Prospective cohort	60	>50% stenosis	All	92	58	41	91
Hamdan et al. [9]	Prospective cohort	115	>50% stenosis	Native	93	73	62	96
				CABG	100	75	95	100
Opolski et al. [10]	Prospective cohort	475	>50% stenosis	All	98	37	67	94
Harris et al. [16]	Retrospective cohort	100	>50% stenosis	All	99	56	86	94
Chieffo et al. [17]	Retrospective cohort	491	–	All	–	–	48	–

Abbreviations: Sn, Sensitivity; Sp, Specificity; PPV, Positive predictive value; NPV, Negative predictive value; CABG, Coronary artery bypass graft.

**Table 2:** Invasive assessment of myocardial ischemia in patients undergoing TAVI.

Study	Study Design	Patients (n)	Reference Standard	Optimal FFR by ROC	Optimal iFR by ROC	Cut-Offs Tested	Sn (%)	Sp (%)	NPV (%)	PPV (%)
Scarsini et al. [36]	Prospective cohort	28	Adenosine SPECT	0.78	–	FFR < 0.78	87	88	92	81
						iFR < 0.82	80	69	86	60
Yamanaka et al. [34]	Prospective cohort	95	Adenosine SPECT	0.82	0.82	–	–	–	–	–
Scarsini et al. [35]	Prospective cohort	252 (85 AS)	FFR ≤ 0.80	–	0.83	iFR < 0.83	72	84	96	78

Abbreviations: FFR, Fractional flow reserve; iFR, instantaneous wave-free ratio; ROC, Receiver operating characteristic; Sn, Sensitivity; Sp, Specificity; NPV, Negative predictive value; PPV, Positive predictive value; SPECT, Single-photon emission computed tomography; AS, aortic stenosis.

**Table 3:** Prognostic impact of CAD in TAVI candidates.

Study	Design	Patients (*n*)	Follow-Up (Months)	Stratification	Outcome(s)	
Lopez Otero et al. [77]	Retrospective cohort	349 (187 CAD)	35.2 (mean)	No CAD	MACE (*p* = 0.91)	39%
				rSS = 0		45%
				0 < rSS < 8		40%
				rSS ≥ 8		47%
Huczek et al. [78]	Registry	896 (462 CAD)	1	No CAD	Mortality (*p* = 0.14)	5.1%
				bSS < 22		8.9%
				bSS ≥ 22		6.9%
Witberg et al. [58]	Retrospective cohort	1270 (453 CAD)	22.8 (median)	No CAD	Mortality (*p* < 0.001)	21.9%
				bSS < 22		26.1%
				bSS ≥ 22		51.9%
				No CAD	Mortality (*p* < 0.001)	21.9%
				rSS < 8		23.2%
				rSS > 8		45.1%
Ryan et al. [79]	Prospective cohort	402 (193 CAD)	12	SS-II < 37.4	MACE (*p* = 0.001)	13.4%
				37.4 ≤ SS-II ≤ 44.0		14.9%
				SS-II > 44.0		31.3%
Shamekhi et al. [80]	Prospective cohort	666 (437 CAD)	24.7 (mean)	No CAD	Mortality (*p* = 0.001)	26.2%
				bSS < 24		34.8%
				bSS ≥ 24		46.8%
				No CAD	Mortality (*p* = 0.01)	25.9%
				rSS ≤ 3		31.4%
				rSS >3		41.5%
				0 < SS-II < 37	Mortality (*p* < 0.001)	27%
				37 ≤ SS-II < 47		23.5%
				47 ≤ SS-II < 55		33.5%
				SS-II ≥ 55		49.2%
Ahad et al. [81]	Retrospective cohort	70 (all CAD)	24	Mean bSS = 29.0	Mortality	31.6%
Paradis et al. [82]	Retrospective cohort	377 (295 CAD)	12	No CAD	MACE (*p* = 0.61)	26.8%
				bSS ≤ 23		23.3%
				23 ≤ bSS ≤ 32		16.7%
				bSS ≥ 33		22.0%
				No CAD	MACE (*p* = 0.01)	26.8%
				rSS < 8		0%
				rSS ≥ 8		10.8%
Manoly et al. [83]	Case series	4 (all CAD)	12 (median)	Mean bSS = 20.6	Mortality	25%
D’Ascenzo et al. [84]	Meta-analysis	8334 (3994 CAD)	12	bSS >22 versus bSS <22	Mortality (*p* = 0.001)	OR 1.71 (1.24–2.36)
				PCI with rSS <8 versus PCI not performed	Mortality (*p* = 0.04)	OR 0.34 (0.12–0.93)
Witberg et al. [85]	Meta-analysis	3107 (1645 CAD)	8.4–36	High rSS versus no CAD	Mortality (*p* < 0.01)	OR 1.85 (1.42–2.40)
				High rSS versus low rSS	Mortality (*p* < 0.001)	OR 1.69 (1.26–2.28)
				Low rSS versus no CAD	Mortality (*p* = 0.33)	OR 1.11 (0.89–1.39)
Chauhan et al. [86]	Retrospective cohort	238 (99 CAD)	14.9 (mean)	bSS ≤ 2	Mortality, MACE, revascularization(*p* = 0.27)	21.3%
				3 ≤ bSS ≤ 10		16.3%
				bSS ≥ 11		21.2%
Penkalla et al. [87]	Prospective cohort	40 (28 CAD)	12 and 60	Mean bSS = 7.6	Mortality	41.4 and 69.6%
Kobayashi et al. [88]	Case series	12 (all CAD)	Intra-hospital	Mean bSS = 22.4	Mortality, MACE	0%
Koskinas et al. [89]	Retrospective cohort	577 (367 CAD)	24	No CAD and cTnT <15 × ULN	Mortality	11.6%
				bSS > 22 and cTnT >15 × ULN		41.1%
Witberg et al. [90]	Registry	287 (49 CAD)	24	No CAD ^a^	MACE (*p* = 0.19 ^a–b^ and *p* = 0.002 ^a–c^)	16.1%
				bSS ≤ 22 ^b^		24.4%
				bSS > 22 ^c^		75%
				No CAD ^a^	MACE (*p* = 0.606 ^a–b^ and *p* = 0.001 ^a–c^)	16.1%
				cSS ≤ 63 ^b^		18.7%
				cSS > 63 ^c^		41.2%
				No CAD ^a^	MACE (*p* = 0.196 ^a–b^ and *p* < 0.001 ^a–c^)	16.1%
				0 < rSS < 9 ^b^		8.6%
				rSS ≥ 9 ^c^		78.6%
O’Sullivan et al. [91]	Registry	108 (80 CAD)	12	Mean bSS = 16.1	Mortality	25.1%
Penkalla et al. [92]	Cohort	593 (308 CAD)	1	No CAD	Mortality (*p* = 0.61)	5.3%
				CAD, no PCI, mean bSS = 5.7		3.9%
				CAD, PCI, mean bSS = 8.0		2.6%
Khawaja et al. [93]	Retrospective cohort	271 (93 CAD)	1 and 12	0 < bSS ≤ 22	Mortality (*p* = 0.007)	5.2 and 23.3%
				22 < bSS ≤ 32		11.1 and 22.2%
				bSS > 33		14.3 and 57.1%
Stefanini et al. [57]	Registry	445 (287 CAD)	12	No CAD	MACE (*p* = 0.016)	12.5%
				0 ≤ bSS ≤ 22		16.1%
				bSS >22		29.6%
				No CAD	MACE (*p* = 0.043)	12.5%
				0 ≤ rSS ≤ 14		16.5%
				rSS > 14		26.3%
Van Mieghem et al. [94]	Prospective cohort	263 (124 CAD)	16 (median)	rSS = 0	Mortality (*p* = 0.85)	20.1%
				rSS > 0		22.6 %
Saia et al. [95]	Prospective cohort	540 (291 CAD)	58 (median)	CR IR	Mortality (*p* = 0.45)	2.9% 4.6%
Kleczynski et al. [96]	Cohort	101	12	CR IR	Mortality (*p* = < 0.001)	7.1% 75%

Abbreviations: bSS, basal Syntax score; CAD, coronary artery disease; CR, complete revascularization; IR, incomplete revascularization; MACE, major adverse cardiac events; rSS, residual Syntax score; SS, Syntax score; PCI, Percutaneous coronary intervention. ^a,b,c,d^, identify the subgroup.

**Table 4:** Timing of PCI in relation to TAVI.

	Advantages	Disadvantages	Preferred Clinical Scenario
PCI before TAVI	– Improve coronary flow, preventing myocardial ischemia during right ventricular paging – Easy coronary cannulation	– Risk of hemodynamic collapse during PCI	– Acute coronary syndrome – Severe ostial and left main stem lesions – Anatomical consideration (type of-valve, valve-in-valve procedure)
Concomitant PCI and TAVI	– Reduce patient discomfort – Avoid second arterial access – Reduce costs of hospitalizations – no delays in case of rescue/bail-out strategies if needed	– Increase volume of dye – Operator fatigue and X-ray exposure	– Normal kidney function – High bleeding risk
PCI post TAVI	– Improve hemodynamic before PCI – Reliable physiological assessment of CAD	– Risk of ischemia during TAVI	– Complex PCI (to avoid hemodynamic instability related to severe AS) – Borderline CAD

Abbreviations: PCI, percutaneous coronary intervention; TAVI, transcatheter aortic valve implantation; AS, aortic stenosis; CAD, Coronary artery disease.
